# Progress of immune checkpoint inhibitors in the treatment of advanced hepatocellular carcinoma

**DOI:** 10.3389/fimmu.2024.1455716

**Published:** 2024-08-09

**Authors:** Tong Liu, Guorui Meng, Shihui Ma, Junqi You, Liang Yu, Risheng He, Xudong Zhao, Yunfu Cui

**Affiliations:** Department of Hepatopancreatobiliary Surgery, the Second Affiliated Hospital of Harbin Medical University, Harbin, China

**Keywords:** hepatocellular carcinoma, immune checkpoint inhibitors, immunotherapy, tumor immune microenvironment, review

## Abstract

Among primary liver cancers, hepatocellular carcinoma is the most common pathological type. Its onset is insidious, and most patients have no obvious discomfort in the early stage, so it is found late, and the opportunity for surgical radical treatment is lost, resulting in a poor prognosis. With the introduction of molecular-targeted drugs represented by sorafenib, patients with middle- and late-stage liver cancer have regained the light of day. However, their therapeutic efficacy is relatively low due to the limited target of drug action, toxic side effects, and other reasons. At this time, the emergence of immunotherapy represented by immune checkpoint inhibitors (ICIs) well breaks this embarrassing situation, which mainly achieves the anti-tumor purpose by improving the tumor immune microenvironment. Currently, ICI monotherapy, as well as combination therapy, has been widely used in the clinic, further prolonging the survival of patients with advanced hepatocellular carcinoma. This article reviews the development of monotherapy and combination therapy for ICIs in advanced hepatocellular carcinoma and the latest research progress.

## 1 Introduction

Primary liver cancer is currently the sixth most common malignant tumor in the world, and its fatality rate is the third highest among all malignant tumors (1). According to the latest statistics from the World Health Organization (WHO), about 760,000 people worldwide die of liver cancer every year, and the trend is still on the rise (2). Hepatocellular Carcinoma (HCC) accounts for 90% of primary liver cancers (in this article, liver cancer refers to HCC only), and surgical treatment is still the first treatment of choice for patients with early-stage HCC, with a 5-year survival rate of about 70%-80% (3). However, the onset of HCC is insidious, most patients are diagnosed with the disease in the middle to late stage, losing the opportunity for radical surgical treatment. In recent years, in the context of precision liver surgery treatment, molecular targeted therapy and immunotherapy have been successively applied to the clinic, bringing light to patients with intermediate and advanced HCC. Since the molecular targeted drugs represented by sorafenib were approved for the treatment of advanced liver cancer in 2007, due to the accumulation of time, they have gradually exposed the problems of limited action targets, toxic side effects, and drug resistance is an urgent need for a new therapeutic modality to break the therapeutic bottleneck of advanced HCC (4–6). Until 2017, the emergence of immunotherapy for treating advanced HCC has opened up a “new world”, which is best represented by immune checkpoint inhibitors (ICIs) (7). ICIs inhibit tumor progression mainly by improving the tumor immune microenvironment and enhancing the anti-tumor properties of immune cells. However, due to the unique immunosuppressive tumor microenvironment of hepatocellular carcinoma, the overall response rate of tumor cells to ICIs is not high, so efforts to improve the immune response rate have been made throughout the development of ICIs for hepatocellular carcinoma (8–10). Currently, ICIs mainly include programmed death-1 (PD-1) antibodies, programmed death-1 ligand (PD-L1) antibodies, and cytotoxic T lymphocyte-associated protein 4 (CTLA-4) antibodies. This article outlines the mechanism of action, history of development, and recent research progress of ICIs in treating advanced HCC, points out the potential challenges they face, and looks forward to the future direction of development.

## 2 ICIs monotherapy

**Figure 1:**
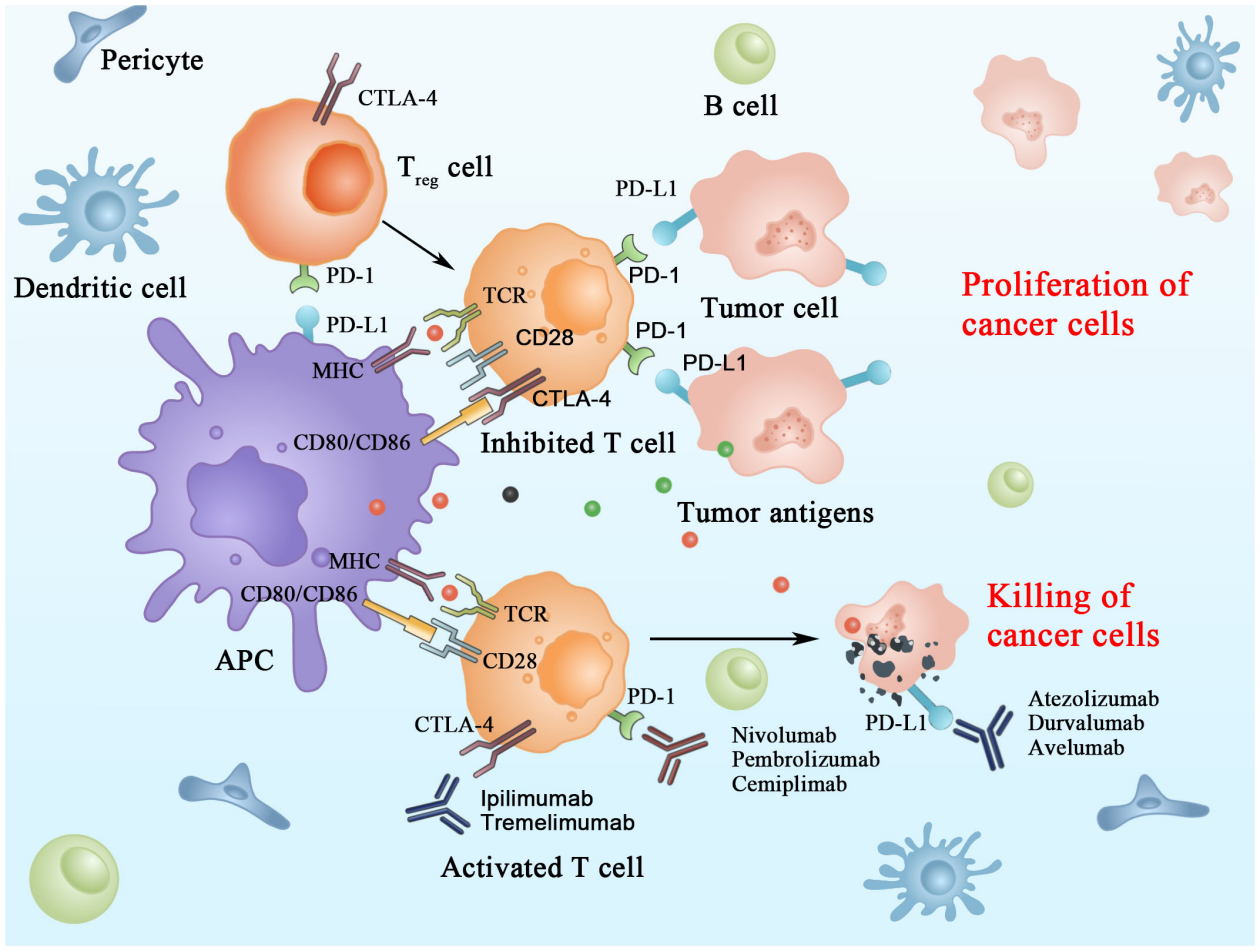
Mechanisms of action of immune checkpoint inhibitors in tumor cells.

The immune checkpoint molecules are inhibitory receptors that trigger immunosuppressive signaling pathways in immune cells. In activating T cells, immune checkpoint receptors transmit co-inhibitory signals that directly suppress the response of T cells; this is considered one of the main mechanisms of immune escape in HCC. ICIs retard tumor growth by blocking IC and thus improving the immunosuppressive microenvironment of tumors in HCC (**Figure 1**) (11).

Currently, the primary study population in clinical trials regarding ICIs is patients with advanced hepatocellular carcinoma. Due to hepatocellular carcinoma’s unique immunosuppressive microenvironment, its overall immune response rate to ICIs is low. Therefore, the primary purpose of ICI research at this stage is to improve this response rate. The completed single-agent clinical trials of ICIs produced satisfactory results, valuable for guiding the clinical treatment of hepatocellular carcinoma (**Table 1**). However, the immune response rate still needs to be improved.

**Table 1:** Outcomes of clinical trials of ICIs monotherapy in HCC.

Monotherapy	Trial name	Phase	Primary endpoint	n	OS, months	PFS, months	ORR, %	TRAE, %	Reference
Nivolumab	Checkmate040 (NCT10658878)	I/II	Safety,ORR, Tolerance	214	15.0	4.1	20	25	(12)
Nivolumab	Checkmate459 (NCT02576509)	III	OS	371/372	16.4/14.7	3.7/3.8	15/7	22/49	(13)
Pembrolizumb	KEYNOTE-224 (NCT02702414)	II	ORR	104	12.9	4.9	17	24	(14)
Pembrolizumb	KEYNOTE-240	III	OS,PFS	273/135	13.9/10.6	3.0/2.8	18.3/4.4	52.7/46.3	(15)
Pembrolizumb	KEYNOTE-394	III	OS	300/153	14.6/13.0	2.6/2.3	12.7/1.3	66.9/49.7	(16)
Camrelizumab	NCT02989922	II	ORR,OS	217	13.8	2.1	14.7	22	(17)
Tislelizumab	RATIONALE-208	II	ORR	249	13.2	2.7	13	15	(18)
Tislelizumab	NCT03412773	III	OS	342/332	15.9/14.1	36.1/11.0	14.3/5.4	96.2/100	(19)
Sintilimab	ChiCTR2000037655	II	PFS	99/99	–	27.7/15.5	–	12.4/-	(20)
Tremelimumab	NCT01008358	II	OS,PFS	20	8.2	6.48	17.6	45	(21)

### 2.1 PD-1 inhibitors

#### 2.1.1 Nivolumab

As a fully humanized IgG4 monoclonal antibody, nivolumab inhibits PD-1 on T-cells’ surface and activates T-cells’ tumor-recognition function to eliminate tumor cells (22, 23). In April 2017, the results of Checkmate040 (NCT01658878), a phase I/II study of nivolumab for the treatment of patients with advanced HCC (n = 262), were published. The results showed that in the dose-expansion arm, the confirmed objective response rate (ORR) was 20%, the median duration of response (mDOR) was 9.9 months, and the median progression-free survival (PFS) was 4.1 months. Meanwhile, in the dose-escalation group, the median survival time (mOS) was 15.0 months, the disease control rate (DCR) was 58%, the ORR was 15%, the mDOR was 17.0 months, and the mPFS was 3.4 months (12). Based on the favorable results of the Checkmate040 trial, nivolumab was first approved by the US Food and Drug Administration (FDA) for second-line treatment of advanced HCC in September of the same year (7). In 2019, the European Society for Medical Oncology (ESMO) published the results of the phase III study Checkmate459 (NCT02576509) of patients receiving nivolumab or sorafenib to treat unresectable HCC. The results showed that compared to sorafenib, nivolumab performed better in terms of overall survival time (OS) and ORR (OS: 16.4 vs. 14.7 months; ORR: 15% vs. 7%) with manageable overall toxicity compared to sorafenib (13). The significance of nivolumab as the opening salvo in HCC immunotherapy for patients with advanced disease can be significant. Although nivolumab does not significantly improve survival time in patients with advanced HCC compared to sorafenib, it offers a new treatment option for patients who cannot undergo targeted therapy with a reliable safety profile, and it also provides a good foundation for other subsequent immunotherapies.

#### 2.1.2 Pembrolizumab

Pembrolizumab is a potent humanized IgG4 monoclonal antibody that targets the immune checkpoint PD-1 and blocks its interaction with ligands, thereby preventing tumor cells from evading anti-tumor immunity (24, 25). In June 2018, the results of KEYNOTE-224 (NCT02702414), a phase II study of pembrolizumab for the treatment of patients with advanced HCC (n = 104), were published. The results showed that pembrolizumab had an ORR of 17%, an mPFS of 4.9 months, and an OS of 12.9 months, with 76 (24%) patients experiencing grade 3 or higher treatment-related adverse events (TRAEs) (14). In November 2018, based on the success of the KEYNOTE-224 trial, pembrolizumab became the second drug after nivolumab to receive FDA approval as a second-line therapy for the treatment of unresectable HCC (26). After KEYNOTE-224, phase III studies on pembrolizumab monotherapy for advanced HCC have been conducted. In June 2019, the American Society of Clinical Oncology (ASCO) was the first to publish the results of KEYNOTE-240, a global phase III study of pembrolizumab for the treatment of patients with advanced HCC (n = 278), which was selected for the primary endpoints of OS and PFS. The results showed an ORR of 18.3%, mOS of 13.9 months, and mPFS of 3.0 months, with 147 patients (52.7%) experiencing grade 3 or higher TRAEs (15). Meanwhile, in Asia, KEYNOTE-394, a randomized, double-masked phase III clinical trial of pembrolizumab in patients with advanced HCC (n = 300), is in full swing. The results of the study, which will be presented at ASCO 2022, showed that patients in the pembrolizumab group had a prolonged OS (14.6 vs. 13.0 months) and increased mPFS (2.6 vs. 2.3 months) and a significantly higher ORR (12.7% vs 1.3%), as compared with patients in the placebo group (16). The discovery of pembrolizumab has added a new therapeutic drug for patients with advanced HCC. It has shown promising efficacy and safety, as nivolumab and pembrolizumab have been put into the clinic one after another, and more and more clinical trials of immunotherapeutic drugs for advanced HCC have also been carried out. For some time, immunotherapy for HCC has become a popular medical research.

#### 2.1.3 Camrelizumab

Camrelizumab, or SHR-1210, is an anti-PD-1 IgG4 monoclonal antibody with potent anti-tumor activity (27, 28). With the rise of HCC immunotherapy, PD-1 inhibitors independently developed by China have been introduced and have achieved good efficacy. In February 2020, the results of a phase II study (NCT02989922) on camrelizumab for treating patients with advanced HCC (n=217) were published. The study showed an ORR of 14.7%, a 6-month overall survival of 74.4%, and grade 3 or 4 TRAEs in 47 patients (22%) (17). In this study, camrelizumab showed promising anti-tumor activity and manageable toxicity. Based on the results of this study, camrelizumab was formally approved by the National Medical Products Administration (NMPA) in March of the same year for the treatment of advanced HCC (29). Camrelizumab is the first PD-1 inhibitor independently developed in China and approved for liver cancer indication in China and the third PD-1 inhibitor globally. The approval of camrelizumab has encouraged China’s pharmaceutical developers and brought numerous benefits to patients with advanced liver cancer. Compared with other imported PD-1 inhibitors, it has a more affordable price and guaranteed efficacy, which marks the arrival of the era of immunotherapy for Chinese liver cancer patients.

#### 2.1.4 Others

As China’s first self-developed PD-1 inhibitor for hepatocellular carcinoma, the launch of camrelizumab has pushed the HCC immunotherapy boom to another wave. Since March 2020, immunotherapeutic drugs independently developed by China, such as tislelizumab, sintilimab, and toripalimab, have been introduced and have shown promising efficacy (30–32). In October 2022, the results of a phase II study (RATIONALE-208) of tislelizumab for treating previously treated patients with advanced HCC (n=249) were reported. The results showed that tislelizumab had an ORR of 13%, a DCR of 53%, and a mOS of 13.2 months, with a total of 38 patients (15%) reporting grade 3 or higher TRAEs, most commonly elevated hepatic transaminases (18). In October 2023, the results of a phase III study (NCT03412773) of tislelizumab in patients with advanced HCC (n=342) were published. Compared with sorafenib, the former showed an overall superiority in mOS, mPFS, and ORR (mOS: 15.9 vs. 14.1 months; mPFS: 36.1 vs. 11.0 months; ORR: 14.3% vs. 5.4%), and the incidence of TRAE was also lower than the latter (96.2% vs. 100%) (19). This result shows that tislelizumab has better anti-tumor activity and safety. In January 2024, the results of a study of sintilimab as adjuvant therapy in resected HCC patients (n=99) with concomitant microvascular invasion (ChiCTR2000037655) were published. The results showed that the mRFS in the sintilimab group was 27.7 months, with 1-year and 2-year survival rates of 99.0% and 87.9%, respectively, and a 12.4% incidence of grade 3 or 4 TRAEs (20). In all of these effective prognostic indicators, the sintilimab group was superior to the active monitoring group, thus demonstrating the effectiveness and safety of sintilimab as a postoperative adjuvant therapy for high-risk HCC patients. There are relatively few clinical studies on these PD-1 inhibitor monotherapies, and it is expected that more clinical studies will be put in place to validate further the efficacy of these PD-1 inhibitor monotherapies in the treatment of advanced HCC.

### 2.2 PD-L1 inhibitors

PD-L1 is one of the ligands for PD-1, also known as B7-H1 or CD274. The expression of PD-L1 is mainly found in tumor cells, Kupffer’s cells, and hepatocytes in HCC (33). As PD-L1 was overexpressed in HCC and combined with PD-1, it inhibited the proliferation and activation of T cells, inactivated T cells. It ultimately led to immune escape, further promoting tumor cell growth (34). Thus, blocking PD-L1 has also emerged as a potential therapeutic strategy for HCC. Currently, two main PD-L1 inhibitors are used for treating advanced HCC, atezolizumab, and durvalumab; both are humanized IgG1 monoclonal antibodies against PD-L1. In June 2020, the results of a phase Ib study (GO30140) on atezolizumab treatment in patients with advanced HCC (n=59) were published. The study showed that the atezolizumab treatment group had an ORR of 17%, mPFS of 3.4 months, and 2 patients (3%) experienced severe TRAEs, which was not as good as the overall outcome of the atezolizumab combined with the bevacizumab treatment group (35). Currently, there are relatively few studies on PD-L1 inhibitor monotherapy for the treatment of advanced HCC, which is still mainly based on combination therapy, and it is expected that more PD-L1 inhibitor monotherapies can be put into clinical studies in the future in order to find a suitable answer.

### 2.3 CTLA-4 inhibitors

CTLA-4, also known as CD152, is a protein receptor that functions to down-regulate T cells (36). CTLA-4 is expressed not only in activated T cells but also in regulatory T cells. It acts as an “off” switch when bound to CD80 or CD86 on the surface of antigen-presenting cells (37). The two main types of CTLA-4 inhibitors commonly used in the clinic today are tremelimumab, a fully humanized IgG2 monoclonal antibody, and ipilimumab, a fully humanized IgG1 monoclonal antibody, both of which can effectively block CTLA-4 binding. In 2013, the European Association for the Study of the Liver (EASL) annual meeting report published the results of a phase II study (NCT01008358) of tremelimumab for the treatment of patients with hepatitis C-associated HCC (n=20). The study demonstrated that tremelimumab had a DCR of 76.4%, an mOS of 8.2 months and that the treatment was generally well tolerated, with a significant reduction in viral load (21). Due to the limited studies on CTLA-4 inhibitor monotherapy for advanced HCC, its anti-tumor activity and safety cannot be accurately assessed at this time, and more studies are expected to follow to validate it further.

## 3 ICIs combination therapy

**Figure 2:**
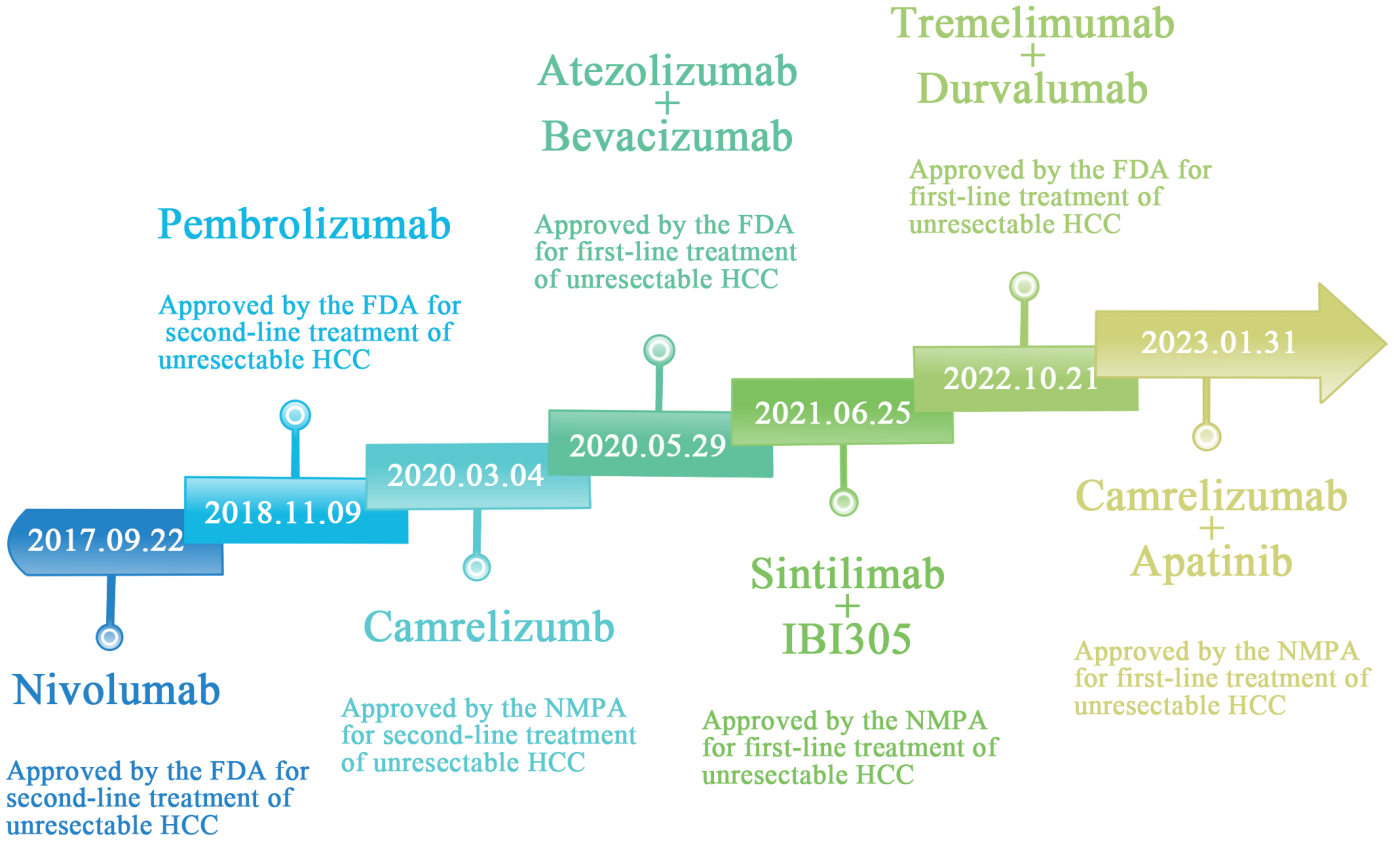
FDA and NMPA approval of immune checkpoint inhibition schedule for unresectable hepatocellular carcinoma.

Although the FDA or NMPA has approved several PD-1/PD-L1/CTLA-4 inhibitor monotherapies for use in advanced HCC, their efficacy is still limited and is not the treatment of choice (**Figure 2**). With further clinical studies on immunotherapy for hepatocellular carcinoma, immune-combination therapy is a better treatment modality for patients with advanced HCC, which can further improve the therapeutic efficacy (**Table 2**) (60–65).

**Table 2:** Outcomes of clinical trials of ICIs combination therapy in HCC.

Combination therapy	Trial name	Phase	Primary endpoint	n	OS, months	PFS, months	ORR,%	TRAE,%	Reference
TACE									
TACE plus SBRT and avelumab	START-FIT (NCT03817736)	II	Proportion of patients who may be cured	33	30.3	21.4	67	33	(38)
TACE plus TKI and camrelizumab	CHiCTR2000039508		PFS,ORR	87	–	10.5	71.3	67.8	(39)
TACE plus Lenvatinib and sintilimab	NCT04599790	II	PFS	30	18.4	8.0	60	40	(40)
RFA									
RFA plus tremelimumab	NCT01853618		OS,PFS	32	12.3	7.4	26.3	–	(41)
RFA plus nivolumab	NIVOLVE (UMIN000026648)	II	RFS	53	–	26.3	–	18.9	(42)
RFA plus pembrolizumb	IMMULAB (NCT03753659)	II	ORR	30	–	–	13.3	–	(43)
RFA plus toripalimab	ChiCTR1900027807		RFS	20/20	–	15.4/8.0	–	–	(44)
RT [^90^Y]									
RT plus nivolumab	CA209-678 (NCT03033446)	II	ORR	36	20.2	27.6	36	14	(45)
RT plus durvalumab	SOLID	I/IIa	TTP	24	–	6.9	83.3	8.7	(46)
RT plus pembrolizumab	HCRNGI15-225 (NCT03099564)		PFS	27	20.3	9.95	30.8	48.1	(47)
HAIC									
HAIC plus lenvatinib and toripalimab	NCT04044313	II	PFS	36	17.9	10.4	63.9	11.1	(48)
HAIC plus camrelizumab and apatinib	NCT04191889	II	ORR	35	–	10.38	77.1	37.1	(49)
Anti-VEGF									
Bevacizumab plus atezolizumab	G030140 (NCT02715531)	Ib	ORR,PFS	104	17.1	12.4	36	–	(35)
Bevacizumab plus atezolizumab	IMbrave150 (NCT03434379)	III	ORR,PFS	336	67.2	6.8	–	56.5	(50)
IBI305 plus sintilimab	ORIENT-32 (NCT03794440)	II/III	Safety,OS, PFS	380/191	-/10.4	4.6/2.8	21/4	14/18	(51)
TKI									
Lenvatinib plus pembrolizumab	NCT03006926	Ib	Safety,Tolerance, ORR,DOR	104	22	8.6	36	67	(52)
Lenvatinib plus pembrolizumab	LEAP-002 (NCT03713593)	III	OS,PFS	395/399	21.2/19.0	8.2/8.0	–	17/17	(53)
Apatinib plus camrelizumab	NCT03092895	II	Safety,Tolerance	28	13.2	3.7	10.7	92.9	(54)
Apatinib plus camrelizumab	CARES-310 (NCT03764293)	III	OS,PFS	272/271	22.1/15.2	5.6/3.7	25/6	88/68	(55)
Lenvatinib plus tislelizumab	NCT04401800	II	ORR	64	–	8.2	38.7	28.1	(56)
ICIs									
Nivolumab plus ipilimumab	NCT03222076	II	Safety,Tolerance	14/13	–	19.53/9.4	–	43/23	(57)
Durvalumab plus tremelimumab	NCT02519348	I/II	Safety	332	18.73	2.17	24	37.8	(58)
Durvalumab plus tremelimumab	HIMALAYA (NCT03298451)	III	OS	393	16.4	3.8	20.1	25.8	(59)

### 3.1 ICIs combined with interventions

Transcatheter arterial chemoembolization (TACE) is an interventional HCC treatment commonly used in treating intermediate and advanced HCC. The chemotherapeutic drugs are delivered directly to the hepatic artery through a catheter. At the same time, the blood supply to the tumor is blocked by using an embolic agent to achieve tumor necrosis and a reduction in its size (66). Using this treatment modality, it is possible to downstage some patients with intermediate to advanced HCC tumors, thus providing the opportunity to achieve radical surgical treatment and prolong survival (67). In addition, TACE can enhance anti-tumor immunity by releasing tumor antigens from killed tumor cells, and immunotherapy can, in turn, strengthen this anti-tumor response, which side-steps the feasibility of TACE in combination with ICIs. Also, TACE has relative limitations, such as low conversion rates, so combination therapy seems more sensible. Several studies have confirmed that ICIs and TACE are efficacious and safe in treating intermediate and advanced HCC (68–70). There is a case report of successful stage reduction of unresectable hepatocellular carcinoma by TACE in combination with tislelizumab, followed by radical surgical resection, with postoperative pathological findings showing complete necrosis of the tumor and no tumor recurrence at 6.0 months postoperatively (71). Based on the promising anti-tumor effects produced by ICIs in combination with interventional therapy, the International Society for Multidisciplinary Interventional Oncology (ISMIO) International Expert Group consensus statement in February 2021 affirmed that TACE combined with the option of systemic therapy regimens can improve the outcome of unresectable HCC (72). In 2023, The Asia-Pacific Primary Liver Cancer Expert Meeting (APPLE) announced the START-FIT (NCT03817736), a phase II study of TACE in combination with stereotactic radiotherapy and avelumab for the treatment of advanced HCC patients (n=33) results. The results showed an ORR of 67%, a DCR of 70%, an mPFS of 21.4 months, an mOS of 30.3 months, and an mDOR of 20.2 months for triple therapy, with 11 (33%) patients experiencing a grade 3 or higher TRAEs, and 4 (12%) patients receiving curative therapy (38). In July of the same year, the results of a study (ChiCTR2000039508) on TACE in combination with TKIs and camrelizumab for treating patients (n=87) with advanced unresectable HCC were published. The results showed an ORR of 71.3%, an mPFS of 10.5 months, and a DCR of 89.7%, as confirmed by mRECIST; ten patients (11.5%) successfully underwent conversion therapy, all achieving R0 resection (39). In April 2024, the results of another phase II study (NCT04599790) of TACE in combination with sintilimab and lenvatinib for advanced HCC (n=30) were published. The results showed an ORR of 60%, mPFS of 8.0 months, DCR of 86.7%, mOS of 18.4 months, and grade 3 or higher TRAEs in 12 patients (40%)

(40). These studies have demonstrated the synergistic anti-tumor effect of TACE combined with ICIs, which may allow unresectable advanced HCC patients to gain access to conversion therapy and prolong survival. In addition, there is also the phase II study of TACE in combination with nivolumab for intermediate-stage HCC (IMMUTACE) (73) and the phase III LEAP-012 (NCT04246177) study of TACE in combination with lenvatinib and pembrolizumab for intermediate stage HCC (74), which have also achieved good results but has not yet clarified the advantageous population receiving TACE combined with ICIs for advanced HCC and the advantages and disadvantages of each combination therapy have not been clarified. More relevant studies will follow to validate the above questions and provide clear answers.

### 3.2 ICIs combined with radiofrequency ablation

Radiofrequency ablation (RFA) therapy is a commonly used local treatment for early-stage HCC, especially for HCC patients with tumor diameters <3 cm is reproducible, minimally invasive, and has low complications (75). RFA treatment activates systemic anti-tumor immune responses and inhibits the immune escape of tumor cells; however, due to the weakness of these responses, they do not allow complete control of the tumor, contributing to the high recurrence rate of RFA (76, 77). Recurrence rates as high as 50% to 70% within 20 to 30 months after successful RFA have been reported, suggesting that single ablative therapy does not appear to be a perfect option for the treatment of early HCC (78). Based on the mechanism by which RFA causes HCC recurrence, it is easy to think of the feasibility of combination immunotherapy. Several studies have demonstrated the synergistic anti-tumor effect of RFA combined with immunotherapy (79–81). In 2017, results from a study (NCT01853618) of the CTLA-4 inhibitor tremelimumab in combination with RFA for advanced HCC (n=32) were published. The study showed that patients treated with the combination had a 6-month progression-free survival time rate of 57.1%, a 12-month progression-free survival time rate of 33.1%, a median OS of 12.3 months, and a significant reduction in viral load in 12 of 14 patients with quantifiable hepatitis C (41). In February 2022, the results of NIVOLVE (UMIN000026648), a phase II study of adjuvant nivolumab after surgical resection/radiofrequency ablation for the treatment of patients with HCC (n=53), were published. The study showed that patients in the combination therapy group had a 1-year RFS of 26.3 months, an 18.9% incidence of grade 3-4 TRAEs, and an overall favorable treatment outcome (42). In February 2023, the results of IMMULAB (NCT03753659), a phase II study of pembrolizumab in combination with radiofrequency ablation for the treatment of patients (n=30) with early-stage HCC, were published. According to RECIST v1.1, the confirmed ORR was 13.3%, which did not meet the provisional mOS (43). In September 2023, the results of a prospective controlled study (ChiCTR1900027807) of toripalimab combined with radiofrequency ablation for treating recurrent HCC were published. The results showed that mRFS was higher in the combination therapy group compared to single RFA treatment (15.4 vs. 8.0 months, HR:0.44, P<0.05) (44). Comprehensive studies on RFA combined with ICIs in recent years have shown that the combination of RFA and ICIs can effectively make up for some of the limitations of RFA, reduce the recurrence rate of tumors, prolong the survival period, and have a controllable safety profile. However, it is not without the lack of persuasiveness due to the small sample size that more multi-center and large-sample studies are expected to further validate the benefits of ICIs in combination with RFA therapy.

### 3.3 ICIs combined with radiotherapy

Radiotherapy is divided into two types: internal radiotherapy and external radiotherapy, which are suitable for patients with advanced HCC, especially those with combined portal vein cancer thrombosis. In recent years, radiotherapy has achieved good results in treating advanced HCC. Among them, selective internal radiotherapy (SIRT) using yttrium [90Y] resin microspheres has been a famous study in HCC in recent years. SIRT injects the radionuclide yttrium [90Y] microspheres containing beta-emitting radionuclides into the tumor tissue via the hepatic artery. Yttrium [90Y] microspheres kill the tumor cells by releasing short-range radiation and cause minimal damage to the normal liver tissue, characterized by a fast onset of action, minimal damage, and precise positioning (82). Yttrium [90Y] was first marketed in Australia in 1998 and has subsequently been used primarily as a palliative treatment for unresectable HCC, and in the last few years, has emerged as a potential down-staging strategy for unresectable hepatocellular carcinoma due to its findings of efficacy in tumor shrinkage and liver hypertrophy (83). In 2022, China’s first yttrium [90Y] resin microsphere intervention, led by Academician Jiahong Dong, was implemented in Boao LeCheng, Hainan, and successfully downstaged a patient with Chinese liver cancer stage (CNLC) IIIa to stage Ia with radical surgical treatment. The tumor cells in the resected specimen were almost entirely necrotic (84). These results demonstrate the feasibility and safety of yttrium [90Y] microspheres for treating advanced HCC. Studies have confirmed that the systemic immune system is activated during radiotherapy, and the combination of ICIs further enhances the therapeutic efficacy and synergistic anti-tumor effect with a reliable safety profile (85, 86). In October 2021, the results of a phase II study CA209-678 (NCT03033446) of radioembolism using yttrium [90Y] resin microspheres in combination with nivolumab for the treatment of patients (n=36) with advanced HCC were published, the primary endpoint of this study was ORR and the secondary endpoint was PFS. The study showed an ORR of 30.6%, mPFS of 20.2 months, and grade 3 or higher TRAEs in 5 patients (14%) (45). In September 2023, the results of SOLID, an I/IIa study of durvalumab in combination with yttrium [90Y] resin microspheres for the treatment of patients (n=24) with locally advanced unresectable HCC, were published. The study showed mPFS of 6.9 months, ORR of 83.3%, DCR of 91.7%, failure to achieve mOS, and grade 3 TRAEs in 2 (8.7%) patients (46). In February 2024, the results of a preliminary study HCRNGI15-225 (NCT03099564) on pembrolizumab in combination with yttrium [90Y] resin microspheres for the treatment of patients (n=27) with advanced HCC were published, showing an mPFS of 9.95 months, an mOS of 20.30 months, an ORR of 30.8% and a DCR of 84.6%, and grade 3 or higher TRAEs occurred in 13 of 27 patients (48.1%) (47). Results have also been published from studies of sintilimab and tislelizumab in combination with radiation therapy, which have shown good efficacy (87, 88). In recent years, the combination of radiotherapy and ICIs has been increasingly used in the treatment of advanced HCC, and its ability to enable some patients to complete tumor downstaging for radical treatment and further prolong the survival of patients has become a hot research topic.

### 3.4 ICIs combined with chemotherapy

Hepatic arterial infusion chemotherapy (HAIC) is one of the primary means of treatment for intermediate and advanced HCC. The primary chemotherapy regimen approved in China is FOLFOX4, which selectively administers chemotherapeutic drugs (including oxaliplatin, fluorouracil, and folinic acid) to the blood-supplying arteries of intrahepatic tumors mainly through an arterial catheter to increase the local concentration, thus exerting a potent anti-tumor effect, and possessing therapeutic characteristics of precise targeting and low toxicity (89–91). Some studies have confirmed that oxaliplatin can induce immunogenic cell death and modulate the tumor cell microenvironment, making oxaliplatin-containing FOLFOX4 chemotherapy regimens combined with ICIs a potential treatment option for unresectable advanced HCC (92). In 2022, the results of a study from China evaluating the efficacy and safety of atezolizumab and bevacizumab in combination with HAIC for the treatment of advanced HCC were published, which enrolled a total of 52 eligible patients with advanced HCC for triple therapy. The results showed an ORR of 67.3%, mPFS of 10.6 months, OS was not achieved, all TRAEs were controlled, and further analysis concluded that extrahepatic metastases were an independent risk factor associated with PFS (93). In the same year, the results of another China’s phase II study (NCT04044313) on the combination of lapatinib and toripalimab with HAIC in patients with advanced HCC (n=36) were also published. The results showed that mPFS was 10.4 months, mOS was 17.9 months, ORR was 63.9%, and mDOC was 14.4 months, with 4 (11.1%) patients experiencing grade 3 or higher TRAEs and no treatment-related deaths (48). In 2023, the results of a phase II study (NCT04191889) on the combination of camrelizumab and apatinib with HAIC in patients with advanced HCC (n=35) were published. The study showed an ORR of 77.1%, DCR of 97.1%, mPFS of 10.38 months, and failure to achieve mOS. A total of 13 patients (37.1%) developed grade 3 or higher TRAEs, and six patients (17.1%) achieved disease downstaging and radical surgery after triple therapy (49). The relevant studies in recent years show that the current combination of ICIs and molecular targeting with HAIC for the treatment of advanced HCC has an excellent synergistic effect, which can further improve the anti-tumor activity and have controllable safety. However, based on the limited number of studies on the combination of the three treatments, more studies are still needed to determine the value of their clinical application.

### 3.5 ICIs combined with targeted therapy

#### 3.5.1 ICIs combined with angiogenesis inhibitors

Prior to the introduction of immunotherapy, molecularly targeted therapies had been the sole therapeutic modality for the systemic treatment of advanced HCC, remaining a monopoly for a decade. Targeted therapies block the growth and proliferation of liver cancer cells by targeting specific signal transduction pathways in liver cancer and adopting a point-to-point approach whereby the drug binds to specific receptors or molecules on the surface of liver cancer cells (94, 95). *Bevacizumab* is an angiogenesis inhibitor, which not only inhibits angiogenesis and thus reduces the blood supply to the tumor but also regulates the tumor’s immune response, a mechanism of action that offers the possibility of subsequent combination with ICIs for the treatment of advanced HCC (96). In 2018, ASCO was the first to publish the results of the phase Ib study GO30140 (NCT02715531) of atezolizumab in combination with bevacizumab (T + A) for the treatment of patients (n=104) with unresectable HCC, the results showed that the combination of the two had a manageable safety profile with a PFS of 12.4 months, a mOS of 17.1 months, an ORR of 36% and a DCR of 71% (35). Due to the synergistic anti-tumor effect of the “T+A” combination regimen shown in the GO30140 study, follow-up studies were soon to follow. In November 2019, ESMO published the results of the phase III study IMbrave150 (NCT03434379) of the “T+A” combination therapy for the treatment of patients (n=336) with unresectable HCC, atezolizumab in combination with bevacizumab showed better DFS and OS rates compared to sorafenib (DFS: 6.8 vs 4.3 months;1-year overall survival rate: 67.2% vs 54.6%) (50). The IMbrave150 further confirms that the combination of the two has good anti-tumor activity in treating advanced HCC. Based on the success of the IMbrave150 study, the FDA and NMPA approved the “T+A” regimen in May and October 2020, respectively, for the treatment of unresectable HCC without prior systemic therapy (97). University societies and guidelines recommend this combination regimen (T + A) as a first-line treatment for advanced HCC (98–103). In October 2020, Cinda Biologics announced the results of ORIENT-32 (NCT03794440), a Phase II-III study of sintilimab in combination with IBI305 (a bevacizumab analog) for the treatment of unresectable HCC. Compared with patients in the sorafenib-treated group, sintilimab in combination with IBI305 significantly improved mPFS and ORR (mPFS: 4.6 vs 2.8 months; ORR: 21% vs 4%), and although sintilimab in combination with IBI305 did not achieve the prespecified mOS, it was still superior to the sorafenib group by 10.4 months (51). Based on the success of the ORIENT-32 trial, in June 2021, the NMPA approved sintilimab in combination with IBI305 as the first-line treatment for advanced HCC. In addition, the results of the phase II study (NCT04843943) on sintilimab in combination with bevacizumab as a conversion therapy for resectable intermediate-stage HCC, led by academician Fan Jia, were presented for the first time at ESMO 2022. The results of the study showed that the ORR and DCR were 23.3% and 90%, respectively, and a total of 13 patients (43.3%) met the criteria for hepatic resection and underwent surgical treatment, after which the patients recovered well and had no recurrence for the time being (104). Currently, ICIs combined with angiogenesis inhibitors have been widely used in intermediate and advanced HCC and have achieved promising therapeutic results. The combination of the two has synergistic solid anti-tumor activity. It can achieve tumor downstaging for intermediate-stage HCC patients with the opportunity to achieve radical surgical treatment and further prolong the patient’s survival.

#### 3.5.2 ICIs combined with tyrosine kinase inhibitors

With the continuous exploration of ICIs in combination with molecular targeted therapy for the treatment of HCC, TKIs in combination with ICIs are feasible and effective in treating advanced HCC. TKIs inhibit the growth and proliferation of tumor cells and promote apoptosis mainly by inhibiting cellular signal transduction (105). Its combination with ICIs has a synergistic anti-tumor effect, further improving the survival of patients with advanced HCC while ensuring safety. In 2019, the ESMO Annual Meeting presented for the first time the results of the phase Ib study (NCT03006926) of pembrolizumab plus lenvatinib for the treatment of patients (n=104) with unresectable HCC. With a confirmed ORR of 36% according to RECIST v1.1, an mDOR of 12.6 months, an mPFS of 8.6 months, and a mOS of 22.0 months, 67 percent of patients experienced grade 3 or higher TRAEs (52). In 2022, the results of the phase III study LEAP-002 (NCT03713593) of pembrolizumab in combination with lenvatinib for the treatment of patients with advanced HCC (n=395) were published, showing a mOS of 21.2 months and an mPFS of 8.2 months, which were both better than in the placebo group but fell short of the pre-determined thresholds (53). At the same time, our researchers have been actively involved and have achieved results that have attracted the world’s attention. In 2021, the results of a phase II study (NCT03092895) of kamrelizumab in combination with apatinib for treating advanced primary hepatocellular carcinoma were published. The study showed an ORR of 10.7 percent, an mPFS of 3.7 months, and an mOS of 13.2 months, with 26 patients experiencing grade 3 or higher TRAEs (54). At the ESMO Annual Meeting 2022, the results of the phase III study CARES-310 (NCT03764293) on kamrelizumab in combination with apatinib for unresectable HCC were presented by Prof Shukui Qin, which showed that compared to sorafenib, the combination therapy had an ORR of 25%, a DCR of 78%, an mOS of 22.1 months and an mPFS of 5.6 months, which were all significantly better than the former (55). Based on the excellent results CARES-310, the CSCO Liver Cancer Guidelines recommended it as a first-line treatment for advanced HCC in the same year (100). In January 2023, the NMPA formally approved the first-line treatment of advanced HCC, achieving a significant breakthrough in treating advanced HCC with ICIs combined with TKIs. In addition, the phase II study of tislelizumab in combination with lenvatinib for treating unresectable HCC (NCT04401800) also achieved good results, showing good anti-tumor activity and tolerability (56). At present, the clinical studies of ICIs combined with small-molecule TKIs for the treatment of advanced HCC have had a series of successive reports, especially the karelizumab combined with apatinib regimen proposed by Prof. Shukui Qin in China, the only large clinical study of ICIs combined with small molecule TKIs for advanced HCC that has obtained positive dual endpoints of OS and PFS to date, adds another reliable treatment option for patients with advanced HCC and has landmark status in the treatment of hepatocellular carcinoma.

### 3.6 Dual-immunity combination therapy

Compared to the limited availability of ICIs as monotherapy, dual-immunity combination therapy achieves “1 + 1>2” efficacy. In October 2020, the results of a Checkmate040 randomized clinical trial were published, which showed better anti-tumor activity and safety in the nivolumab combined with the ipilimumab treatment group compared to monotherapy (106). In January 2022, results from a phase II study (NCT03222076) of nivolumab in combination with ipilimumab for resectable HCC were published, with the combination having superior mPFS (19.5 vs 9.4 months) and a manageable overall safety profile compared to nivolumab monotherapy (57). The above study blocked both PD-1 and CTLA-4 immune checkpoints, which further inhibited the immune escape of tumor cells and delayed tumor growth. In July 2021, the results of a phase I/II study (NCT02519348) of durvalumab plus tremelimumab in the treatment of patients with unresectable HCC (n=332) were published. The study showed an ORR of 24%, an mOS of 18.73 months, an mPFS of 2.17 months, and an incidence of TRAEs of grade 3 or higher of 38.7% with the combination of both treatments (58). In 2022, the results of HIMALAYA (NCT03298451), a phase III study of durvalumab plus tremelimumab for treating unresectable HCC, were published. The study results showed an ORR of 20.1%, mPFS of 3.8 months, and mOS of 16.4 months. Subsequent follow-up observation found that the 3-year overall survival rate and 4-year overall survival rate of the combination therapy group were 30.7% and 25.2%, respectively, which were significantly higher than those of the sorafenib group, which were 19.8% and 15.1% (59, 107). Based on the success of the HIMALAYA study, the FDA formally approved tremelimumab in combination with durvalumab for the first-line treatment of unresectable HCC in October 2022 (108). This is the first time a PD-L1 inhibitor and a CTLA-4 inhibitor have been combined, making this regimen the second FDA-approved first-line regimen for treating advanced HCC after “T+A”,which will benefit more patients.

## 4 Conclusions and perspectives

Due to the high metastasis and recurrence of liver cancer, the global mortality rate of liver cancer patients has remained high every year. The emergence of immunotherapy represented by ICIs has brought light to patients with advanced liver cancer and, at the same time, broken the monopoly of molecular targeted therapy for advanced liver cancer, opening up a new pattern of liver cancer treatment. ICIs inhibit tumor growth and proliferation by blocking immune checkpoints, thereby inhibiting the immune escape of tumor cells from immune cells, significantly increasing the overall survival and disease-free survival of patients and improving the quality of their survival. Compared with the limited efficacy of PD-1/PD-L1/CTLA-4 inhibitor monotherapy, immuno-combination therapy has better efficacy for patients with advanced HCC, and some patients can even achieve tumor downstaging and radical surgical treatment through combination therapy. Although immunotherapy has benefited many liver cancer patients, it still faces many challenges, such as drug resistance during treatment, the advantageous population suitable for each combination therapy, which is still unclear, and the occurrence of immune-related adverse events after treatment. Significantly, since the vast majority of liver cancer patients in China develop from hepatitis B, finding ICI monoclonal antibodies that are more suitable for our patients has become significant. In addition, it has been confirmed that ICI treatment can reactivate the hepatitis virus in a small number of patients, resulting in fulminant hepatitis or even liver failure, and how to eliminate this phenomenon is also a subsequent problem to be solved. All these issues need to be validated by ongoing multi-center, large-sample, prospective controlled studies and in-depth clinical and basic research to individualize treatment. In addition to this, immunotherapy should not be limited to immune checkpoint inhibitors, but relay cell therapy and tumor vaccines have also been the subject of immunological research on advanced liver cancer in recent years, and the results of these studies are also worth looking forward to. Due to the complexity of the pathogenesis and the high degree of malignancy of hepatocellular carcinoma, it is essential to clearly understand that although immunotherapy offers a ray of hope for patients with advanced HCC, ultimately, the efficacy of drug treatment is limited, earlier detection and diagnosis is the top priority, and this requires an in-depth study of the pathogenesis of liver cancer, and then make targeted prevention. We look forward to an early breakthrough in this research direction to reduce the incidence and mortality of liver cancer.
